# Risk factors and *SCN5A*-H558R polymorphism for atrial fibrillation in Tibetans living at different altitudes

**DOI:** 10.1097/MD.0000000000031778

**Published:** 2022-11-18

**Authors:** Renfang An, Jiang Liu, Jinwei Zhang, Fengcai Yao, Dekuan Tian, Fuli Liang, Wenqiang Li, Delian Li, Yiqi Wang, Sai Yan, Qijuan Yang, Yajie Zhang, Xiaoling Su

**Affiliations:** a Department of Cardiology, Qinghai Provincial People’s Hospital, Xining, China; b Department of Cardiac Function, Xi’an No. 03 Hospital, the Affiliated Hospital of Northwest University, Xi’an, Shaanxi, China; c Department of Cardiology, Nanyang Center Hospital, Nanyang, China; d Department of Cardiology, The First Hospital of Xining City, Xining, China; e Department of Cardiology, Qinghai Provincial Hospital of Cardiovascular and Cerebrovascular Diseases, Xining, China; f Graduate School of Qinghai University, Xining, China.

**Keywords:** atrial fibrillation, different elevations, genetic polymorphism, risk factors, *SCN5A*-H558R

## Abstract

Several studies have found associations of genes with atrial fibrillation (AF), including *SCN5A-H558R*. However, there are limited data of these associations among populations living at different altitudes. We investigated the relationship between the *SCN5A-H558R* polymorphism and AF in Tibetans living at different altitudes in Qinghai, China. General clinical and genotype data were obtained from 72 patients with AF and 109 non-AF (NAF) individuals at middle altitudes, and from 102 patients with AF and 143 NAF individuals at high altitudes. Multifactor logistic regression was performed to determine associations and AF risk factors. *SCN5A*-H558R genotypes differed significantly between the AF and NAF groups (*P *< .0125) and the G allele was an independent AF risk factor (*P *< .05) at both altitudes, with no significant differences according to altitude (*P > *.0125). At middle altitudes, age, red blood cell distribution width (RDW-SD), left atrial internal diameter (LAD), and G allele were independent AF risk factors. At high altitudes, age, smoking, hypertension, RDW-SD, free triiodothyronine, LAD, and G allele were independent AF risk factors (*P *< .05). The G allele of *SCN5A*-H558R might be an independent risk factor of AF both high and middle altitude, but there are some differences in other clinical risk factors of AF.

## 1. Introduction

Atrial fibrillation (AF) is the most common type of intraventricular arrhythmia, characterized by rapid and disordered atrial electrical activity. Epidemiological studies have suggested that the overall AF incidence has increased over time, and its prevalence is expected to increase further.^[1]^ Accordingly, the medical and health burden for treating AF-associated complications such as heart failure and stroke is expected to increase.^[2]^ Although there are diverse treatment strategies for AF, the exact pathogenesis has not yet been fully elucidated. Recently, the relationship between genes and AF has attracted increasing attention, and specific genotypes have been reportedly associated with a higher risk of AF development.^[3–5]^

*SCN5A* encodes the α subunit of the human cardiac sodium channel NAv1.5, which regulates gating, cellular localization, intracellular trafficking, and degradation to affect cardiac excitability and conduction. *SCN5A* mutations are associated with various arrhythmic syndromes, including AF, sick sinus syndrome, long QT syndrome, Brugada syndrome, conduction disease, atrial arrest, mixed arrhythmia phenotype overlap syndrome, and drug arrhythmias.^[6]^ *SCN5A*-H558R is a loss-of-function mutation associated with reduced sodium current density and a shortened refractory period, decreased atrial conduction velocity, and increased susceptibility to atrial arrhythmias. This mutation also plays a role in the maintenance of AF, thus increasing the risk of AF development.^[7]^ As we all know the *SCN5A*-H558R polymorphism is significantly associated with AF incidence; however, the distribution of the gene frequencies differs significantly among different groups.^[8]^ It may be related to the racial inheritance and living habits of different populations. The Tibetans of Qinghai live in the northeast of the Qinghai-Tibet Plateau, with a strong and persistent living environment pressure caused by cold, dry, low pressure, hypoxia and strong ultraviolet radiation. Some studies at plateau have found some differences in diseases at different altitudes and in different ethnic groups,^[9,10]^ and genetic polymorphisms associated with AF is different between high altitude and plain populations.^[11]^ To the best of our knowledge, differences in the *SCN5A*-H558R polymorphism in populations living at different altitudes have not been reported. Therefore, the aim of this study was to analyze the *SCN5A*-H558R polymorphism in patients with AF living at different altitudes and to better understand the risk factors affecting AF in these patients.

## 2. Materials and methods

### 2.1. Study subjects

Between June 2018 and August 2021, we enrolled 72 patients with AF and 109 non-AF (NAF) individuals living at 1800 to 2500 m (middle altitude), and 102 patients with AF and 109 NAF individuals living at 3500 to 4500 m (high altitude).

The inclusion criteria of AF group were as follows: an AF diagnosis by 24-hours dynamic echocardiogram (ECG) or 12-lead ECG, Tibetan ethnicity, aged between 18 and 85 years, with no immediate or extended family history of heterogamy. The exclusion criteria were congenital heart disease, cardiomyopathy, chronic pulmonary heart disease, chronic obstructive pulmonary disease, severe liver and kidney dysfunction, blood disorders, rheumatic immune diseases, hyperthyroidism, hypothyroidism, infectious diseases, or malignant tumors. The non-AF group was hospitalized in the cardiology department without developing AF, and the exclusion criteria were the same as the AF group

This study was conducted according to the principles stated in the Declaration of Helsinki and was reviewed by the Medical Ethics Committee of Qinghai Provincial People’s Hospital (Xining, China). Before collecting blood samples, written informed consent was obtained from all patients.

### 2.2. Data collection

Patient information, including sex, age, ethnicity, smoking history, drinking history, as well as the medical history of hypertension, diabetes, and coronary heart disease, was recorded.

Approximately 3 mL of peripheral blood was collected at fasting in Y tubes to extract DNA, determine the genotype, and examine relevant blood indices. Laboratory examinations included white blood cell count, red blood cell (RBC) count, hemoglobin, red blood cell distribution width (RDW-SD), platelet count (Plt), fasting blood glucose, triglyceride, total cholesterol (TC), high-density lipoprotein, low-density lipoprotein (LDL cholesterol), uric acid (UA), thyroid-stimulating hormone (TSH), free triiodothyronine (FT3), and free thyroxine.

The study participants also underwent cardiac color Doppler ultrasound, which included measurements of left atrial internal diameter (LAD), ventricular septal thickness, left ventricular posterior wall thickness, and left ventricular ejection fraction (LVEF).

### 2.3. DNA sequencing

DNA was extracted by professionals of the molecular pathology laboratory of Qinghai Provincial People’s Hospital (Beijing Yaanda Biotechnology Co., Ltd., catalog number: DP348). DNA purity and concentration were determined using a Boao microspectrophotometer (NanoQ Micro, Beijing Boao Jingdian Biotechnology Co., Ltd.). DNA sequencing was performed by Xi’an Zhenpin Biotechnology Co., Ltd. to identify relevant DNA sites. The following primers were used: SCN5A-H558R-Forward primer, 5’-GCCAGTGGCACAAAAGACAGGCT-3’; SCN5A-H558R-Reverse primer, 5’-GGAACTGCTGATCAGTTTGGGAGA-3’.

### 2.4. Statistical analysis

The SPSS statistical analysis software version 21.0 (IBM, Armonk, NY) was used for data analysis. The count data are expressed as a composition ratio; the chi-square test was used for comparisons between 4 groups, and chi-square segmentation was used for pairwise comparisons. Data with a normal distribution are expressed as mean; analysis of variance was used for multiple group comparisons, and the least-significant difference *t* test was used for pairwise comparisons. Data with a skewed distribution are expressed as median and interquartile range (IQR); the Kruskal–Wallis *H* test was used for intergroup comparisons, and pairwise comparisons between groups were performed after the *H* test. The Hardy–Weinberg equilibrium for genotype was evaluated using a chi-square test. Binary logistic regression was used to identify independent risk factors for AF. *P *< .05 was considered statistically significant.

## 3. Results

### 3.1. General participant information

Age significantly differed among all 4 subgroups (*P *< .05). Moreover, a significant difference was observed between the middle-altitude AF and NAF groups (*P *< .05), between the high-altitude AF and NAF groups (*P *< .05), and between the middle- and high-altitude NAF groups (*P *< .05) (Table 1).

**Table 1:** Comparison of general data M(P25, P75).

Index	Middle altitude		High altitude		*H*	*P*
	NAF group	AF group	NAF group	AF group		
Age	53.0 （45.5，62.5)♦	65.0 （59.3，75.0)*	59.0 （50.0，72.0)	72.0 （65.0，75.0)♦	84.127	<.001

AF = atrial fibrillation, NAF = non-atrial fibrillation.

*Comparison with the middle altitude NAF group, *P* < 0.05; ♦Comparison with the high altitude NAF group, *P*<0.05.

No significant differences were observed between the middle-altitude AF and NAF groups or between the middle- and high-altitude AF groups in any of the measured parameters (*P *> .0125). Significant differences were noted in smoking status and hypertension occurrence between the high-altitude AF and NAF groups (*P *< .0125), as well as in terms of smoking status and incidence of diabetes mellitus and coronary heart disease between the middle- and high-altitude NAF groups (*P *< .0125) (Table 2).

**Table 2:** General data comparison (%).

Index		Middle altitude		High altitude		χ^2^	*P*
		NAF group	AF group	NAF group	AF group		
Sex	Male	76(69.7)	44(61.1)	81(56.6)	49(48.0)	10.668	.014
	Female	33(30.3)	28(38.9)	62(43.4)	53(52.0)		
Smoking		42(38.5)♦	20(27.8)	26(18.2)	42(41.2)♦	19.285	<.001
Drinking		33(30.3)	15(20.8)	27(18.9)	13(12.7)	10.324	.016
Hypertension		44(40.4)	42(58.3)	45(31.5)	65(63.7)♦	30.858	<.001
Diabetes mellitus		37(33.9)♦	14(19.4)	24(16.8)	29(28.4)	11.734	.008
Coronary heart disease		22(20.2)♦	17(23.6)	10(7.0)	16(15.7)	13.500	.004

AF = atrial fibrillation, NAF = non-atrial fibrillation.

*Comparison with the middle altitude NAF group, *P* < .0125; ♦Comparison with the high altitude NAF group, *P* < .0125; ※Comparison with the high altitude AF group, *P* < .0125.

### 3.2. General laboratory parameters

Significant differences were observed in RDW-SD, Plt, TC, LDL cholesterol, and UA between the middle-altitude AF and NAF groups (*P *< .05). Similarly, there were significant differences in RDW-SD, Plt, TC, high-density lipoprotein cholesterol, LDL cholesterol, and FT3 between the high-altitude AF and NAF groups (*P *< .05). Additionally, significant differences were observed in RBC and UA between the middle- and high-altitude NAF groups (*P *< .05). However, there were no significant differences between the middle- and high-altitude AF groups (*P *> .05) (Table 3).

**Table 3:** Comparison of the general laboratory indicators M(P25, P75).

Index	Middle altitude		High altitude		*H*	*P*
	NAF group	AF group	NAF group	AF group		
WBC（×10^9^/L）	5.69 （4.75,7.01)	5.69 （4.34,7.34)	6.11 （5.05,7.50)	5.90 （5.08,7.33)	4.827	.185
RBC（×10^12^/L）	5.01 （4.49,5.49)^ ♦^	4.72 （4.36,5.67)	5.35 （4.85,5.84)	5.09 （4.54,5.83)	13.623	.003
Hb（g/L）	154.00（138.00,168.00)	153.00（137.50,165.00)	157.00 （141.00,176.00)	154.50（132.75,171.00)	3.320	.345
RDW-SD（fL）	44.60 （41.75,48.55)	49.40 （44.43,56.20)^ *^	43.00 （40.50,47.20)	47.65 （44.00,54.55)^♦^	53.307	<.001
Plt（×10^9^/L）	186.00 （145.50,238.00)	152.00 （121.25,203.25)^ *^	207.00 （163.00,254.00)	155.50（110.75,209.25)^♦^	36.760	<.001
FBG（mmol/L）	4.98 （4.46,7.39)	5.09 （4.60,7.11)	4.93 （4.51,6.27)	4.92 （4.41,5.82)	1.946	.584
TG（mmol/L）	1.21 （0.90,1.69)	1.08 （0.82,1.33)	1.23 （0.93,1.63)	1.07 （0.77,1.52)	11.448	.010
TC（mmol/L）	4.12 （3.46,4.95)	3.02 （2.61,4.07)^ *^	4.24 （3.55,4.97)	3.35 （2.66,4.11)^♦^	65.974	<.001
HDL（mmol/L）	1.01 （0.79,1.22)	0.92 （0.73,1.10)	0.99 （0.83,1.20)	0.87 （0.70,1.02)^♦^	21.445	<.001
LDL（mmol/L）	2.52 （1.82,3.05)	1.95 （1.37,2.43)^ *^	2.67 （2.10,3.27)	2.07 （1.64,2.64)^♦^	45.971	<.001
UA（μmol/L）	312.00 （256.50,399.00)^ ♦^	384.5（311.75,454.25)^ *^	383.00（285.0,444.00)	375.50 （314.00,508.25)	21.498	<.001
TSH（mIU/L）	2.88 （1.63,4.62)	2.72 （1.07,3.79)	2.61 （1.90,3.55)	2.29 （1.59,3.10)	7.654	.054
FT4（pmol/L）	11.35 （10.03,12.81)	12.19（10.95,13.90)	12.02（10.19,14.68)	12.47 （10.22,16.03)	12.447	.006
FT3（pmol/L）	4.49 （4.12,5.01)	4.85 （4.35,5.29)	4.36 （3.74,4.90)	4.58 （4.21,5.12)^♦^	20.396	<.001

AF = atrial fibrillation, FBG = fasting blood glucose, FT3 = free triiodothyronine, FT4 = free thyroxine, HB = hemoglobin, HDL cholesterol = high-density lipoprotein, LDL cholesterol = low-density lipoprotein, NAF = non-atrial fibrillation, Plt = platelet count, RBC = red blood cell count, RDW = red blood cell distribution width, TG = triglyceride, TSH = thyroid-stimulating hormone, UA = uric acid, WBC = white blood cell count.

*Comparison with the middle altitude NAF group, *P* < .05; ♦Comparison with the high altitude NAF group, *P* < .05; ※Comparison with the high altitude AF group, *P* < .05.

### 3.3. Cardiac doppler ultrasound indicators

There were significant differences in LAD between the middle-altitude AF and NAF groups (*P *< .05). Similarly, LAD and LVEF differed significantly between the high-altitude AF and NAF groups (*P *< .05). However, the indicators between the middle- and high-altitude NAF groups (*P > *.05) or between the middle- and high-altitude AF groups were comparable (*P *> .05) (Table 4).

**Table 4:** Comparison of cardiac doppler ultrasound indicators M(P25, P75).

Index	Middle altitude		High altitude		*H*	*P*
	NAF group	AF group	NAF group	AF group		
LAD（mm）	35.00（32.00,38.00)	45.00 （41.00,48.75) *	35.00 （31.00,40.00)	42.00 （38.00,48.00)♦	118.114	<.001
IVST（mm）	10.00 （9.00,11.00)	10.00 （9.00,11.75)	10.00 （9.00,12.00)	11.00 （10.00,13.00)	10.544	.014
LVPWT（mm）	10.00 （9.00,11.00)	10.00 （9.00,11.00)	10.00 （9.00,11.00)	10.00 （9.00,12.00)	4.948	.176
LVEF（%）	64.00 （60.00,67.00)	62.00 （56.00,67.00)	63.00 （60.00,67.00)	60.00 （55.75,63.78)♦	22.746	<.001

AF = atrial fibrillation, IVST = ventricular septal thickness, LVEF = left ventricular ejection fraction, LVPWT = left ventricular posterior wall thickness, NAF = non-atrial fibrillation.

*Comparison with the middle altitude NAF group, *P* < .05; ♦Comparison with the high altitude NAF group, *P* < .05; ※Comparison with the high altitude AF group, *P* < .05.

### 3.4. SCN5A-h558r polymorphism

**Figure 1.**
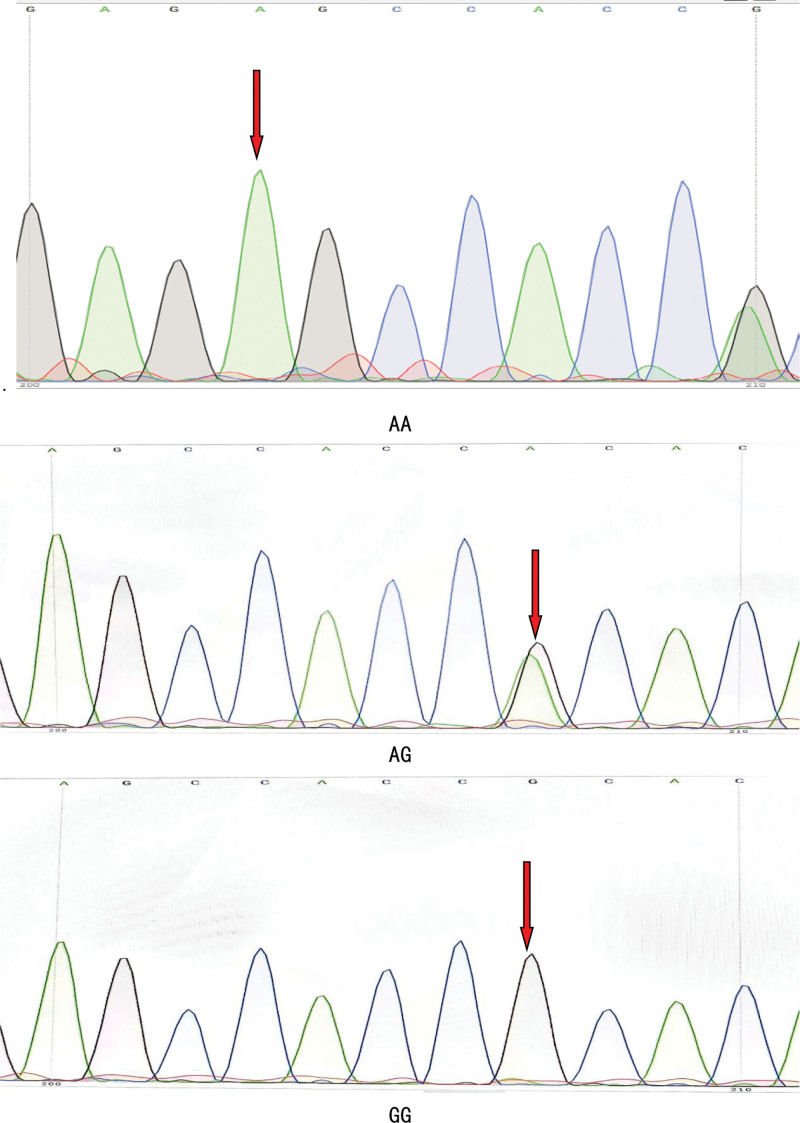
Gene sequence plots.

The genotype frequencies of *SCN5A*-H558R of the 4 groups were consistent with the Hardy–Weinberg equilibrium law with identity (*P* > .05). The genotype and allele distribution frequency of the *SCN5A*-H558R polymorphism differed significantly between the middle-altitude AF and NAF groups (*P *< .0125) and between the high-altitude AF and NAF groups (*P *< .0125). The genotype and allele distribution frequency of the *SCN5A*-H558R polymorphism were not significantly different between the middle- NAF and high-altitude NAF groups (*P *> .0125) or between the middle- and high-altitude AF groups (*P *> .0125) (Fig. 1) (Table 5).

**Table 5:** Comparison of genotype and allele of SCN5A-H558R（%）.

Group		Genotype			H-W		Allele	
		AA	AG	GG	χ^2^	*P*	A	G
Middle altitude	NAF-group	72(66.1)	34(31.2)	3(2.8)	0.183	.913	178(81.7)	40(18.3)
	AF-group	34(47.2)	28(38.9)	10(13.9) *	1.125	.570	96(66.7)	48(33.3) *
High altitude	NAF-group	99(69.2)	39(27.3)	5(3.5)	1.805	.406	237(82.9)	49(17.1)
	AF-group	53(52.0)	35 (34.3)	14(13.7) ♦	3.927	.140	141 (69.1)	63 (30.9) ♦
	χ^2^	23.440					23.615	
	*P*	.001					<.001	

AF = atrial fibrillation, H-W = Hardy-Weinberg balance test, NAF = non-atrial fibrillation.

*Comparison with the middle altitude NAF group, *P* < .0125; ♦Comparison with the high altitude NAF group, *P* < .0125.

### 3.5. Risk factors in the middle-altitude AF group

The index of significant differences between the middle-altitude AF and NAF groups was used as an independent variable and AF was used as the dependent variable for binary logistic regression analysis. The results showed that in the middle-altitude group, age, RDW-SD, LAD and G allele significantly affected AF occurrence (*P *< .05) (Table 6).

**Table 6:** Logistic regression analysis of the risk factors for middle altitude presence of AF.

Variable	b	sb	Wald	*P*	OR(95%CI)
Age	0.078	0.019	16.131	<0.001	1.081 (1.041, 1.123)
RDW-SD	0.061	0.030	4.120	.042	1.063 (1.002, 1.127)
LAD	0.167	0.038	19.764	<.001	1.182 (1.098, 1.273)
G allele	0.852	0.428	3.966	.046	2.344 (1.014, 5.421)
Constant	−16.469	2.709	36.951	<.001	<0.001

AF = atrial fibrillation, LAD = left atrial internal diameter, RDW = red blood cell distribution width.

### 3.6. Risk factors in the high-altitude AF group

The index of significant differences between the high-altitude AF and NAF groups was used as the independent variable and AF was used as the dependent variable for binary logistic regression analysis. The results showed that in the high-altitude group, age, smoking, hypertension, RDW-SD, FT3, LAD and G allele significantly affected AF occurrence (*P *< .05) (Table 7).

**Table 7:** Logistic regression analysis of the risk factors for high altitude presence of AF.

Variable	b	sb	Wald	*P*	OR(95%CI)
Age	0.042	0.014	8.761	.003	1.043(1.014，1.073)
Smoking	1.372	0.399	11.850	.001	3.943(1.806，8.612)
Hypertension	1.122	0.353	10.094	.001	3.070(1.537，6.133)
RDW-SD	0.053	0.026	4.251	.039	1.054(1.003，1.108)
FT3	0.800	0.258	9.640	.002	2.224(1.343，3.685)
LAD	0.108	0.027	16.184	<.001	1.114(1.057，1.174)
G allele	1.072	0.369	8.438	.004	2.921(1.417，6.021)
Constant	−13.886	2.413	33.123	<.001	<0.001

AF = atrial fibrillation, FT3 = free triiodothyronine, LAD = left atrial internal diameter, RDW = red blood cell distribution width.

### 3.7. Risk prediction model

**Figure 2.**
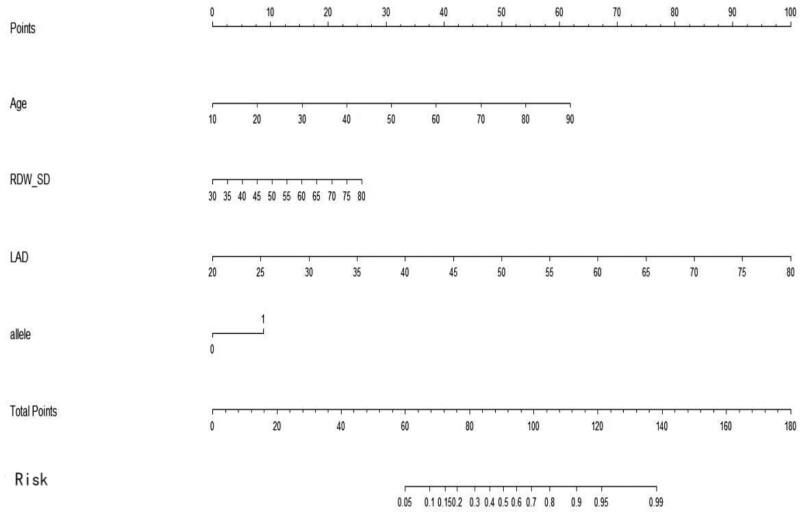
Nomogram of middle altitude（0 means A, 1 means G）.

**Figure 3.**
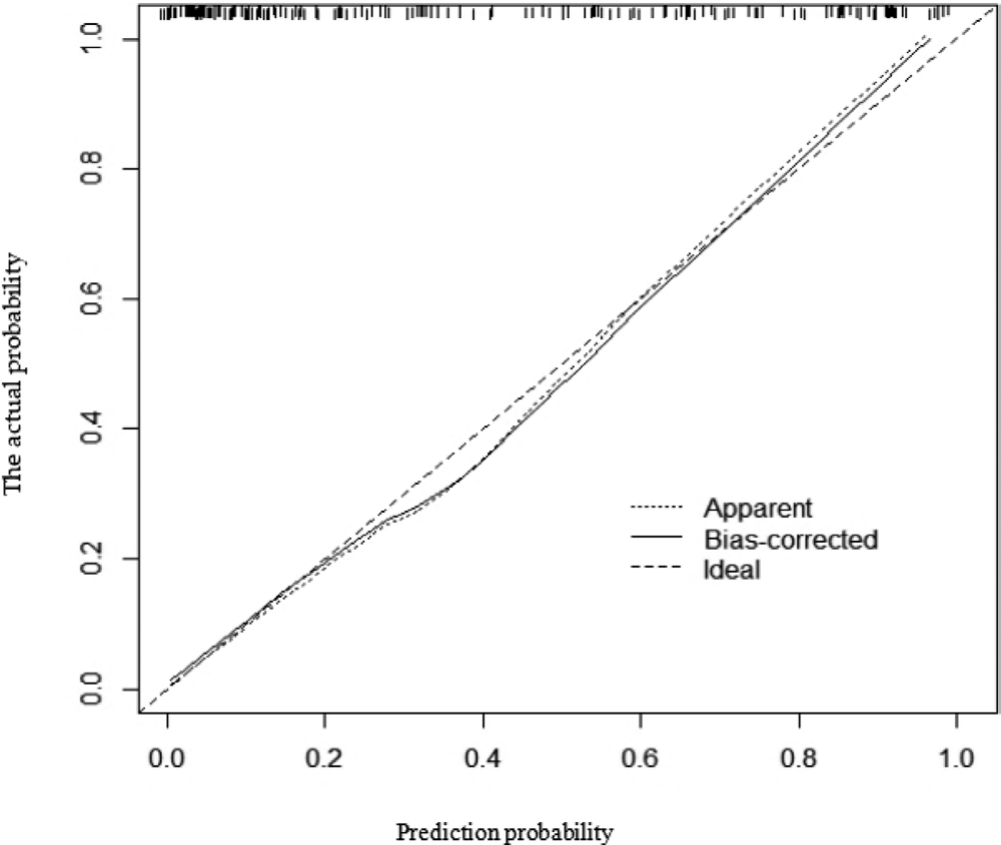
Nomogram of high altitude（Smoking: 0 means no, 1 means yes; hypertension: 0 means no, 1 means yes; allele: 0 means A, and 1 means G）.

**Figure 4.**
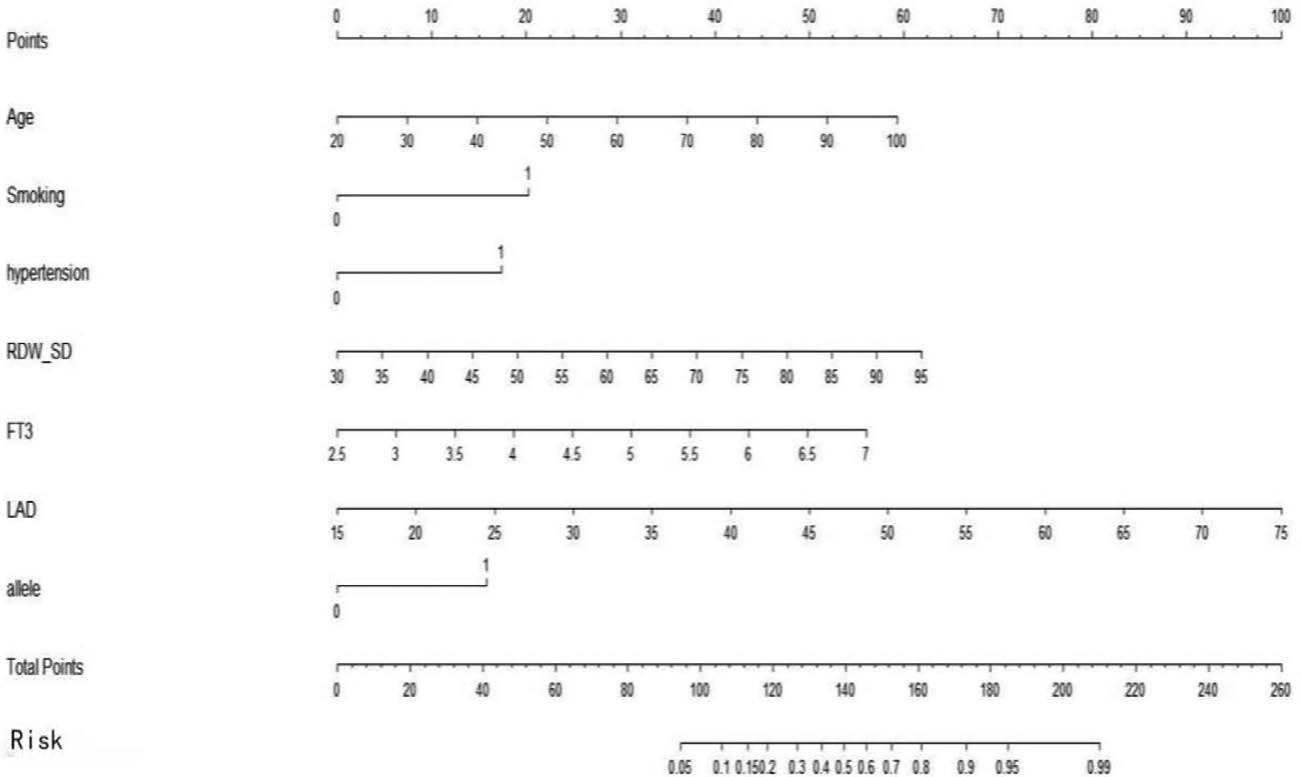
Calibration plot.

**Figure 5.**
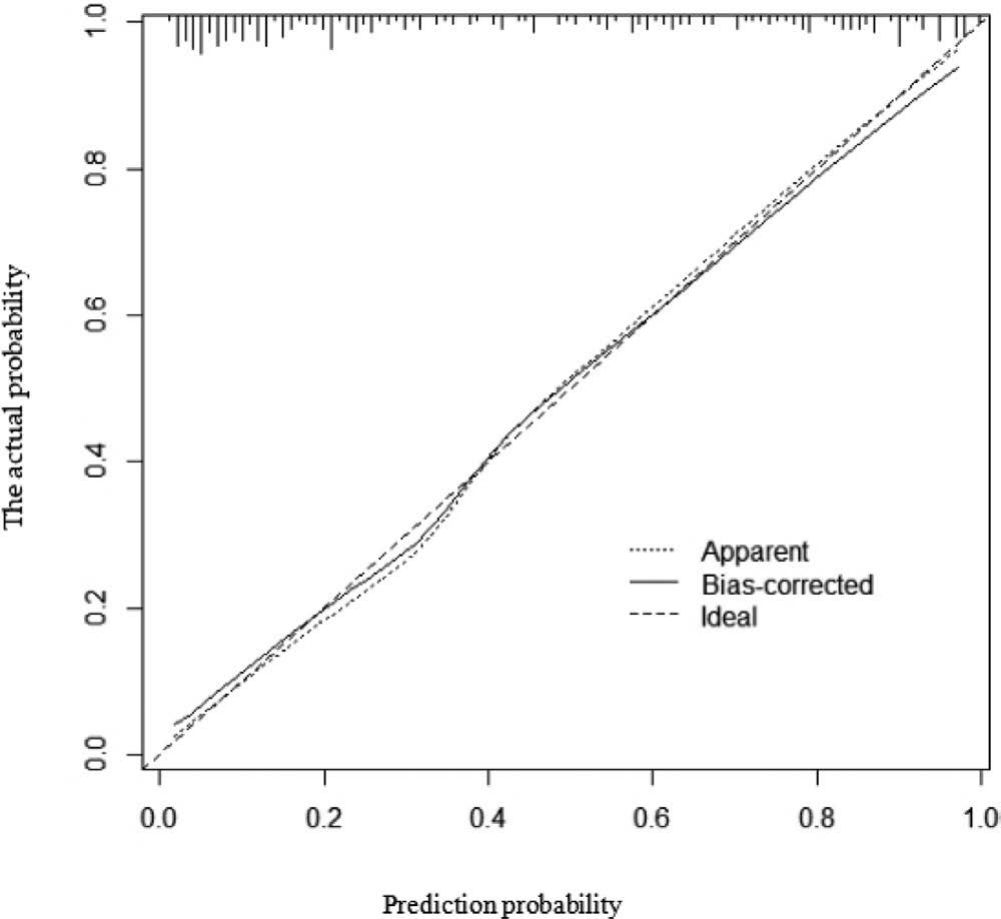
Calibration plot.

Moreover, we integrated the above significant factors into nomograms for the prediction of probability of occurrence of AF at middle (Fig. 2) and high altitudes (Fig. 3), respectively. The probability of developing AF was predicted according to the scoring system corresponding to the nomograph (Tables 8–11). The results suggested that the score of the population at middle altitudes was 91 and that of the population at high altitudes was 140, with a probability of AF of 50%. The prediction accuracy (cindex) of the above scoring system was 0.898 (95% CI: 0.850–0.946) and 0.881 (95% CI: 0.837–0.925) for the population at middle and high altitudes, respectively. The accuracy of the above scoring system was verified according to the calibration plot (Figs. 4 and 5).

## 4. Discussion

AF is a clinically and genetically heterogeneous disease. Both acquired^[12]^ and genetic^[13]^ risk factors have been identified for AF. Multiple studies have confirmed that the susceptibility of AF is related to *SCN5A* mutations. H558R, a common loss-of-function polymorphism of *SCN5A*, results in a T-to-C transition, replacing histidine with arginine in the protein sequence. This changes the functions of the cardiac sodium channel and reduces sodium influx, thereby shortening the action potential time range and increasing the AF risk.^[14]^ The *SCN5A*-H558R polymorphism is reportedly more common in patients with early-onset AF lacking traditional risk factors^[15]^ than in controls, and the allele imparts a 1.6-fold increased risk of isolated AF occurrence. Chen et al^[16]^ reported that the H558R polymorphism plays an important role in increasing AF sensitivity, and the TC and CC genotypes are significantly associated with an increased risk of AF. Yellken et al^[8]^ reported that in the Han and Uyghur populations, AF was associated with the *SCN5A*-H558R polymorphism, and the G allele was an independent risk factor for AF. Additionally, significant differences were noted in the H558R polymorphism between different ethnic groups, suggesting that the relationship between this polymorphism and susceptibility to AF may differ among different populations.

Here, we found that the *SCN5A*-H558R polymorphism was more common in patients with AF than in the NAF group, regardless of altitude. The G allele frequency was also significantly higher in the AF group than in the NAF group (*P* < .0125), indicating its association with the AF occurrence in the Tibetan population in Qinghai. Furthermore, logistic regression analysis showed that the G allele was an independent risk factor for AF occurrence in the Tibetan population regardless of altitude. Early identification of AF susceptibility in a population carrying the G allele may help to regulate other controllable AF risk factors to delay disease progression, thereby reducing AF occurrence.

As the R558 allele may decrease the baseline cardiac sodium current density, it may increase the sensitivity to sodium channel block arrhythmias when patients with AF are treated with class I antiarrhythmic drugs.^[15]^ Therefore, analyzing the relationship between the H558R polymorphism and AF occurrence is important for guiding the individualized use of antiarrhythmic drugs.

Different geographical environments and lifestyle habits at various altitudes may affect the genotype and phenotype and their association. Qinghai Tibetans have lived under low oxygen pressure (hypoxia) conditions, which may alter the occurrence and development of certain diseases when compared with those of the lowland population.^[17–19]^ However, in our study, the genotype and allele frequencies of *SCN5A*-H558R did not differ significantly between the AF and NAF groups at different altitudes; this finding should be verified using a larger cohort in the future. Our study shows that at both medium and high altitudes, the genotype and allele frequencies were significantly different between the AF and NAF groups, with the mutant genes more common in the AF group. The statistical results suggest a clear correlation between the *SCN5A*-H558R gene polymorphism and the occurrence of AF in this region. After binary logistic regression analysis of the AF risk factors with significant differences, the results showed that G allele was a strong independent risk factor of AF. Early identification of susceptible individuals carrying the G allele could help to prevent other controllable AF risk factors to delay and reduce the occurrence of AF. Moreover, with the continued development of genetic molecular technology, these findings may also provide therapeutic targets for the upstream treatment of AF. In addition, carrying the susceptible allele may affect the baseline heart sodium current density. Since the susceptibility gene affects channel function, antiarrhythmic drugs such as class I antiarrhythmic drugs that inhibit sodium ion channels may increase the sensitivity of sodium channel block arrhythmia, leading to the occurrence of other arrhythmic events. Therefore, analyzing the relationship between the *SCN5A*-H558R gene polymorphism and AF in this region also plays an important role in guiding the individualized use of antiarrhythmic drugs in patients with AF.

Moreover, age is a well-known risk factor for AF.^[20,21]^ The prevalence of AF increases with increasing age; in China, the prevalence of AF in adults aged < 55 years was reported to be 0.1%, compared to 9% in those aged 80 years.^[22]^ The lifetime risk of AF increases significantly in individuals aged ≥ 75 years.^[2,23]^ Consistently, age was identified as an independent risk factor for AF at both middle and high altitudes in our study. We hypothesize that the effect of age on AF may be related to age-specific myocardial remodeling, an increase in reactive oxygen species production, and decreased antioxidation capacity. These age-related physiological changes can affect the expression of certain genes and activate specific factors to promote atrial fibrosis, ultimately increasing the AF risk.^[24]^

Smoking can also increase the AF risk.^[12,25,26]^ In our study, smoking was an independent risk factor for AF in the high-altitude group only. In a large cohort study, the AF incidence was 9.5% in smokers and was 7.8% in nonsmokers, suggesting that smoking may be associated with an increased risk of AF.^[27]^ However, this association was less significant after adjusting for cardiovascular risk factors. The authors suggested that this may be due to smoking-related exacerbation of cardiovascular risk factors associated with AF, such as increased oxidative stress, inflammation, and atrial fibrosis. At high altitudes, the hemodynamic load and elevated sympathetic stress can cause oxidative stress, and hemodynamic overload can also trigger an inflammatory response, leading to atrial electrical and structural remodeling, providing a theoretical basis for the occurrence and development of AF. Thus, we considered that the association of smoking with AF at high altitudes might be attributed to the above-mentioned causes. Moreover, nicotine reportedly exerts certain cardiovascular effects.^[28]^ However, a direct link between smoking and AF pathogenesis has not been reported.

The pathological changes associated with hypertension affect the electrical activity of the myocardium, eventually leading to AF development.^[29]^ The Framingham Heart Study^[30]^ showed that both sustained increases in systolic pressure and prolonged antihypertensive treatment were associated with an increased risk of AF. A study in China has shown that highlanders (especially the working class) have a higher prevalence of hypertension.^[31]^ Moreover, the lack of hypertension treatment and control rates due to poor health awareness may explain why hypertension significantly affects AF at high altitudes. Therefore, measures to control the risk factors for hypertension and regulate blood pressure would effectively reduce AF occurrence.

Elevated levels of RDW are often associated with impaired erythropoiesis and degradation. Oxidative stress and inflammation can inhibit the maturation of erythrocytes, and the accumulation of immature erythrocytes will lead to a significant increase in RDW levels in the blood. Studies suggest that higher levels of RDW may reflect an increased risk of AF, and that RDW may serve as an independent predictor of AF after cardiac surgery or others surgery.^[32,33]^ In another study comparing individuals living at different altitudes,^[34]^ RDW was also suggested as an independent risk factor for the occurrence of AF. RDW-SD was suggested as a risk factor for AF at both middle and high altitudes. However, another study showed no obvious difference in RDW of Tibetans living at different altitudes in this region,^[35]^ which is consistent with our results.

Hyperthyroidism is associated with a significantly increased AF risk. A large cohort study^[36]^ reported that AF risk depends on TSH and free thyroxine levels; a decrease in TSH levels increases the AF risk. However, AF risk is lower in patients with overt and subclinical hypothyroidism, suggesting that hypothyroidism may protect against AF. A survey study reported that the normal T3, T4, FT3, and TSH levels of healthy Tibetan adults in Lhasa are slightly lower than those of individuals living in the plain areas.^[37]^ It is worth noting that the Tibetan individuals living in the hypoxic environment of the plateau may have evolved a reduced adaptive thyroid function, which can help them to maintain a low metabolic rate and avoid excessive metabolic consumption. In this study, the results showed that FT3 was a risk factor affecting AF occurrence in the high-altitude group, suggesting that the high FT3 levels may have more pronounced effects on the risk of AF occurrence at high altitudes.

The Framingham Heart Study^[38]^ reported that increased LAD and left ventricular posterior wall thickness and decreased LVEF predict the risk of non-valvular AF. Additionally, these echocardiographic indicators can provide important information on AF risk factors; for instance, the LAD of patients with heart failure combined with AF is significantly higher than that of patients with heart failure combined with sinus rhythm.^[39]^ Our results suggest that LAD affects AF independently in Tibetans at middle and high altitudes, and increased LAD is associated with an increased AF risk. An increase in LAD is also reportedly associated with alcohol consumption,^[40]^ age, male sex, higher body mass index, higher systolic blood pressure, and lower diastolic blood pressure.^[41]^ Unique lifestyle habits and the generally high blood pressure in our study population may have led to increased LAD levels, thus increasing the AF risk. Increased LAD is also associated with an increased risk of mortality in other cardiovascular diseases.^[42]^ Therefore, early prevention and delayed atrial remodeling by actively regulating LAD-associated factors may have important implications in AF and other heart diseases.

This study had certain limitations. The number of Tibetans living in middle-altitude areas is far lower than that in high-altitude areas; thus, the sample size was limited during the study period. In addition, the design of our study only included altitude observation groups at middle and high altitudes, lacking a low-altitude group.

This study indicates that the *SCN5A*-H558R gene polymorphism and other traditional risk factors significantly influence the occurrence of AF. Logistic regression analysis combined with risk factors showed that several risk factors, including the G allele, independently influenced the occurrence of AF. Using the nomogram diagram to obtain a relevant scoring system, based on the corresponding score of each risk factor value, the total number was summed to derive the probability of final AF, which can help to identify the probability of developing AF in people with susceptibility factors. Prediction of disease occurrence by specific theoretical values will have important implications for the early identification and active treatment of AF-susceptible populations in this region.

## 5. Conclusion

We found that AF occurrence in Tibetans at middle and high altitudes in Qinghai is associated with the *SCN5A*-H558R polymorphism. The G allele significantly increases the risk of AF in these individuals. However, we did not find differences in the *SCN5A*-H558R polymorphism between individuals living at different elevations. However, we also identified differences in some traditional AF risk factors associated with different altitudes in Tibetans in Qinghai.

**Table 8:** The scoring system corresponding to the above nomogram is as follows.

Factor		Score	Factor		Score
Age	10	0	LAD	20	0
	20	8		25	8
	30	15		30	17
	40	23		35	25
	50	31		40	33
	60	39		45	42
	70	46		50	50
	80	54		55	58
	90	62		60	67
RDW-SD	30	0		65	75
	35	3		70	83
	40	5		75	92
	45	8		80	100
	50	10	Allele	A	0
	55	13		G	9
	60	15			
	65	18			
	70	21			
	75	23			
	80	26			

LAD = left atrial internal diameter, RDW = red blood cell distribution width.

**Table 9:** The probability of AF corresponding to the above scoring system is as follows.

Normo score	Probability of AF
60	5.00%
68	10.00%
72	15.00%
76	20.00%
82	30.00%
86	40.00%
91	50.00%
95	60.00%
99	70.00%
105	80.00%
113	90.00%
121	95.00%
138	99.00%

AF = atrial fibrillation.

**Table 10:** The scoring system corresponding to the above nomogram is as follows.

Factor		Score	Factor		Score
Age	20	0	FT3	2.5	0
	30	7		3.0	6
	40	15		3.5	12
	50	22		4.0	19
	60	30		4.5	25
	70	37		5.0	31
	80	44		5.5	37
	90	52		6.0	44
	100	59		6.5	50
Smoking	0	0		7.0	56
	1	20	LAD	15	0
Hypertension	0	0		20	8
	1	17		25	17
RDW-SD	30	0		30	25
	35	5		35	33
	40	10		40	42
	45	14		45	50
	50	19		50	58
	55	24		55	67
	60	29		60	75
	65	33		65	83
	70	38		70	92
	75	43		75	100
	80	48	Allele	A	0
	85	52		G	16
	90	57			
	95	62			

FT3 = free triiodothyronine, LAD = left atrial internal diameter, RDW = red blood cell distribution width.

**Table 11:** The probability of AF corresponding to the above scoring system is as follows.

Normo score	Probability of AF
94	5.00%
106	10.00%
113	15.00%
118	20.00%
127	30.00%
133	40.00%
140	50.00%
146	60.00%
153	70.00%
161	80.00%
173	90.00%
185	95.00%
210	99.00%

AF = atrial fibrillation.

## Acknowledgments

We really thank the Qinghai Provincial Department of Science and Technology and the Qinghai Provincial Health and Health Committee for their financial support for this institute.

## Author contributions

CAll authors have read and agreed to the published version of the manuscript.

**Conceptualization:** Renfang An, Jiang Liu.

**Data curation:** Renfang An, Jiang Liu, Jinwei Zhang, Wenqiang Li.

**Formal analysis:** Renfang An, Jiang Liu.

**Investigation:** Jinwei Zhang, Fengcai Yao, Dekuan Tian, Fuli Liang, Wenqiang Li.

**Methodology:** Renfang An, Jiang Liu.

**Project administration:** Delian Li, Yiqi Wang, Sai Yan, Qijuan Yang, Yajie Zhang.

**Resources:** Fengcai Yao, Dekuan Tian, Fuli Liang, Delian Li, Yiqi Wang, Sai Yan, Qijuan Yang, Yajie Zhang.

**Software:** Jinwei Zhang, Wenqiang Li.

**Supervision:** Xiaoling Su.

**Validation:** Xiaoling Su.

**Writing – original draft:** Renfang An, Jiang Liu.

**Writing – review & editing:** Xiaoling Su.

## References

[1] Lane D-A Skjoth F Lip GYH. Temporal trends in incidence, prevalence, and mortality of atrial fibrillation in primary care. J Am Heart Assoc. 2017;6:e005155. 28455344 10.1161/JAHA.116.005155 PMC5524079

[2] Guo Y Tian Y Wang H. Prevalence, incidence, and lifetime risk of atrial fibrillation in China: new insights into the global burden of atrial fibrillation. Chest. 2015;147:109–19. 24921459 10.1378/chest.14-0321

[3] Darbar D Kannankeril P-J Donahue B-S. Cardiac sodium channel (SCN5A) variants associated with atrial fibrillation. Circulation. 2008;117:1927–35. 18378609 10.1161/CIRCULATIONAHA.107.757955 PMC2365761

[4] Xiong H Yang Q Zhang X. Significant association of rare variant p.Gly8Ser in cardiac sodium channel beta4-subunit SCN4B with atrial fibrillation. Ann Hum Genet. 2019;83:239–48. 30821358 10.1111/ahg.12305 PMC6815221

[5] Liu X Li Y Zhang H. The research of ion channel-related gene polymorphisms with atrial fibrillation in the Chinese Han population. Mol Genet Genomic Med. 2019;7:e835. 31270966 10.1002/mgg3.835 PMC6687643

[6] Wilde AAM Amin A-S. Clinical spectrum of SCN5A mutations: long QT syndrome, brugada syndrome, and cardiomyopathy. JACC Clin Electrophysiol. 2018;4:569–79. 29798782 10.1016/j.jacep.2018.03.006

[7] Li W Yin L Shen C. SCN5A variants: association with cardiac disorders. Front Physiol. 2018;9:1372. 30364184 10.3389/fphys.2018.01372 PMC6191725

[8] Yerkenick S. Correlation study between the polymorphism at the H558R locus of the SCN5A gene and the Uygur, Han Chinese atrial fibrillation patients in the Xinjiang region. Xinjiang Medical University, 2017.

[9] Su X Zhou B Wang R. Blood CGRP and ADM content in Tibetan and Han hypertensive patients at different elevations. J Qinghai Med Coll. 2011;32:121–123127.

[10] Zhang W Li X Chen Y. Correlation between essential hypertension and apolipoprotein E gene polymorphism in Han, Tibetan and Hui ethnic groups at high-altitude areas. China Circ Mag. 2007;22:263–6.

[11] Yang Z Ju C Chen H. Analysis of the distribution of CYP2C9 and VKORC1 gene polymorphisms in Han population in alpine regions. Int J Lab Med. 2018;39:2507–10.

[12] Benjamin E-J Levy D Vaziri S-M. Independent risk factors for atrial fibrillation in a population-based cohort. The Framingham Heart Study. JAMA. 1994;271:840–4. 8114238

[13] Sinner M-F Ellinor P-T Meitinger T. Genome-wide association studies of atrial fibrillation: past, present, and future. Cardiovasc Res. 2011;89:701–9. 21245058 10.1093/cvr/cvr001 PMC3039249

[14] Ellinor P Nam E-G Shea M-A. Cardiac sodium channel mutation in atrial fibrillation. Heart Rhythm. 2008;5:99–105. 18088563 10.1016/j.hrthm.2007.09.015

[15] Chen L-Y Ballew J-D Herron K-J. A common polymorphism in SCN5A is associated with lone atrial fibrillation. Clin Pharmacol Ther. 2007;81:35–41. 17185997 10.1038/sj.clpt.6100016 PMC1933493

[16] Chen L Zhang W Fang C. Polymorphism H558R in the human cardiac sodium channel SCN5A gene is associated with atrial fibrillation. J Int Med Res. 2011;39:1908–16. 22117993 10.1177/147323001103900535

[17] A-V Signore SJ. Biochemical pedomorphosis and genetic assimilation in the hypoxia adaptation of Tibetan antelope. Sci Adv. 2020;6:b5447. 10.1126/sciadv.abb5447 PMC7299627 32596473

[18] Chapman M-A Hiscock S-J Filatov D-A. Genomic divergence during speciation driven by adaptation to altitude. Mol Biol Evol. 2013;30:2553–67. 24077768 10.1093/molbev/mst168 PMC3840311

[19] Peng Y Yang Z Zhang H. Genetic variations in Tibetan populations and high-altitude adaptation at the Himalayas. Mol Biol Evol. 2011;28:1075–81. 21030426 10.1093/molbev/msq290

[20] Schnabel R-B Yin X Gona P. 50 year trends in atrial fibrillation prevalence, incidence, risk factors, and mortality in the Framingham Heart Study: a cohort study. Lancet. 2015;386:154–62. 25960110 10.1016/S0140-6736(14)61774-8 PMC4553037

[21] Murphy N-F Simpson C-R Jhund P-S. A national survey of the prevalence, incidence, primary care burden and treatment of atrial fibrillation in Scotland. Heart. 2007;93:606–12. 17277353 10.1136/hrt.2006.107573 PMC1955558

[22] Chien K-L Su T-C Hsu H-C. Atrial fibrillation prevalence, incidence and risk of stroke and all-cause death among Chinese. Int J Cardiol. 2010;139:173–80. 19046608 10.1016/j.ijcard.2008.10.045

[23] Li L-H Sheng C-S Hu B-C. The prevalence, incidence, management and risks of atrial fibrillation in an elderly Chinese population: a prospective study. BMC Cardiovasc Disord. 2015;15:31. 25953603 10.1186/s12872-015-0023-3 PMC4427946

[24] Yan J Thomson J-K Zhao W. The stress kinase JNK regulates gap junction Cx43 gene expression and promotes atrial fibrillation in the aged heart. J Mol Cell Cardiol. 2018;114:105–15. 29146153 10.1016/j.yjmcc.2017.11.006 PMC5800987

[25] Heeringa J Kors J-A Hofman A. Cigarette smoking and risk of atrial fibrillation: the Rotterdam Study. Am Heart J. 2008;156:1163–9. 19033014 10.1016/j.ahj.2008.08.003

[26] Zuo H Nygard O Vollset S-E. Smoking, plasma cotinine and risk of atrial fibrillation: the Hordaland Health Study. J Intern Med. 2018;283:73–82. 28940460 10.1111/joim.12689

[27] Ahmad-M I Mosley C-D O’Neal W-T. Smoking and risk of atrial fibrillation in the REasons for Geographic and Racial Differences in Stroke (REGARDS) study. J Cardiol. 2018;71:113–7. 28886993 10.1016/j.jjcc.2017.07.014 PMC5735021

[28] Benowitz N-L Burbank A-D. Cardiovascular toxicity of nicotine: implications for electronic cigarette use. Trends Cardiovasc Med. 2016;26:515–23. 27079891 10.1016/j.tcm.2016.03.001 PMC4958544

[29] Gumprecht J Domek M Lip GYH. Invited review: hypertension and atrial fibrillation: epidemiology, pathophysiology, and implications for management. J Hum Hypertens. 2019;33:824–36. 31690818 10.1038/s41371-019-0279-7

[30] Rahman F Yin X Larson M-G. Trajectories of risk factors and risk of new-onset atrial fibrillation in the framingham heart study. Hypertension. 2016;68:597–605. 27512109 10.1161/HYPERTENSIONAHA.116.07683 PMC4982514

[31] Zhang Z Wang D Zhao L. Analysis of hypertension prevalence and influencing factors in high-altitude residents. Chin Public Health. 2009;25:1131–2.

[32] Weymann A Ali-Hasan-Al-Saegh S Sabashnikov A. Prediction of new-onset and recurrent atrial fibrillation by complete blood count tests: a comprehensive systematic review with meta-analysis. Med Sci Monit Basic Res. 2017;23:179–222. 28496093 10.12659/MSMBR.903320 PMC5439535

[33] Shao Q Korantzopoulos P Letsas K-P. Red blood cell distribution width as a predictor of atrial fibrillation. J Clin Lab Anal. 2018;32:e22378. 29315856 10.1002/jcla.22378 PMC6817116

[34] Han K Su X Liu J. Red cell distribution width as a novel marker for different types of atrial fibrillation in low and high altitude. Cardiol Res Pract. 2019;2019:6291964. 30984423 10.1155/2019/6291964 PMC6431478

[35] Deng Y Xiaofeng M Wang H. Comparison of the correlation between coronary heart disease and red BC physiological index changes in highland Tibetan and Han populations. Lingnan Cardiovasc Dis J. 2019;25:131–7.

[36] Selmer C Olesen J-B Hansen M-L. The spectrum of thyroid disease and risk of new onset atrial fibrillation: a large population cohort study. BMJ. 2012;345:e7895. 23186910 10.1136/bmj.e7895 PMC3508199

[37] Peng H Li S Wang H. Investigation of normal thyroid hormone values in healthy Tibetan adults in plateau areas. Highland Med J. 2006;03:45–6.

[38] Vaziri S-M Larson M-G Benjamin E-J. Echocardiographic predictors of nonrheumatic atrial fibrillation. The Framingham Heart Study. Circulation. 1994;89:724–30. 8313561 10.1161/01.cir.89.2.724

[39] Zhu N Chen H Zhao X. Left atrial diameter in heart failure with left ventricular preserved, mid-range, and reduced ejection fraction. Medicine (Baltim). 2019;98:e18146. 10.1097/MD.0000000000018146 PMC6890319 31770253

[40] McManus D-D Yin X Gladstone R. Alcohol consumption, left atrial diameter, and atrial fibrillation. J Am Heart Assoc. 2016;5:e004060. 27628571 10.1161/JAHA.116.004060 PMC5079048

[41] McManus D-D Xanthakis V Sullivan L-M. Longitudinal tracking of left atrial diameter over the adult life course: clinical correlates in the community. Circulation. 2010;121:667–74. 20100973 10.1161/CIRCULATIONAHA.109.885806 PMC2823068

[42] Lee H-L Hwang Y-T Chang P-C. A three-year longitudinal study of the relation between left atrial diameter remodeling and atrial fibrillation ablation outcome. J Geriatr Cardiol. 2018;15:486–91. 30364811 10.11909/j.issn.1671-5411.2018.07.005 PMC6198265

